# Trait Architecture of Fruit-Panicle Economic Traits in *Idesia polycarpa* and Its Implications for Breeding

**DOI:** 10.3390/plants15111631

**Published:** 2026-05-26

**Authors:** Kunmin Xiang, Ying Hu, Bangshuang Liu, Yongbin Ou, Bohan Fu, Lijun Wang, Yinan Yao

**Affiliations:** College of Life Sciences and Agri-Forestry, Southwest University of Science and Technology, Mianyang 621010, China

**Keywords:** fruit weight, *Idesia polycarpa*, number of branches, oil content, panicle, panicle density

## Abstract

*Idesia polycarpa* is an important woody oil tree species in East Asia, but the phenotypic framework of fruit-panicle economic traits remains unclear. This study clarified the hierarchical structure of fruit-panicle yield formation and its relationships with panicle density and oil-related traits for breeding. All traits showed continuous variation. Panicle length, primary branch number, fruit stalk length, and fruit size were approximately normally distributed, whereas fresh panicle weight, fruit number per panicle, and secondary branch number were strongly right-skewed. Secondary branch number further showed a typical zero-inflated long-tail pattern. Single-panicle yield was directly determined by fruit number and hundred-fruit weight, while fruit number was jointly regulated by primary and secondary branching. Primary branches formed the basic panicle framework, whereas secondary branches contributed disproportionately to fruit-bearing capacity and extreme heavy-panicle phenotypes. Thus, secondary branch number is the key upstream structural target trait controlling large-panicle formation, while fruit number and hundred-fruit weight are the two major direct yield components. Panicle density is positively correlated with fruit size and branching architecture, and negatively correlated with fruit stalk length, indicating that overly compact panicles may compromise harvest performance by increasing fruit rot risk. Oil content and fatty-acid traits showed substantial variation but were only weakly correlated with most yield-related traits, indicating that high yield and high oil content can be improved simultaneously.

## 1. Introduction

*Idesia polycarpa* Maxim. is an important woody oil-bearing plant widely distributed in East Asia [1,2,3]. Its fruits can be used to produce *I. polycarpa* fruit oil (IPFO), which is regarded as an industrial drying oil due to its high proportion of unsaturated fatty acids. That is, it readily undergoes oxidative polymerization in air to form a solidified film, thereby possessing certain industrial application value [4,5,6]. In addition, because it is rich in phenolic compounds, it can serve as an important raw material for the cosmetics, food, and pharmaceutical industries [7,8,9]. Since IPFO was approved as a novel edible food in 2019, the development and utilization of this species as a woody oil tree have received increasing attention [4,6].

The germplasm resources of *I. polycarpa* are mainly derived from elite individuals selected from wild populations, and its domestication and cultivation history remains short [1,6]. Recent studies and industrial practice have shown that yield (panicle weight) and oil content vary greatly among different accessions of *I. polycarpa* [4,10,11], indicating that both yield and oil content should be considered key breeding targets. In addition, excessive panicle compactness can reduce ventilation and increase fruit rot risk during harvest, making compactness another important evaluation trait [12,13,14]. Although existing evaluation standards describe panicle length, panicle width, and fruit size [4], systematic studies on the composition of economic panicle traits in *I. polycarpa* and their relevance to harvest remain insufficient. This limits the identification of clear breeding target traits and subsequent QTL-associated phenotypic indicators [1,6].

Gramineous crops with typical panicle architecture, such as rice (*Oryza sativa*), have established a mature paradigm for yield formation based on a “branching–grain number–yield” framework [15,16,17]. Specifically, structural traits, including the number of primary branches, the number of secondary branches, the number of grains per branch (spikelet number), and grain size (thousand-grain weight), collectively determine panicle yield. On this basis, researchers have identified key genes and regulatory networks controlling panicle architecture in rice and wheat [18,19].

Similarly, in grape (*Vitis vinifera*) and other crops with a typical cluster architecture, yield-related traits are usually described at three major hierarchical levels: the cluster main axis formed by the central rachis, primary branches, and spikelets (sometimes also referred to as secondary or higher-order branches) [12,14]. On the other hand, grape clusters also have an important trait—cluster density. Overly compact clusters exhibit poor ventilation and light penetration, which favors disease development and can cause uneven berry coloration due to berry-to-berry compression [13,14]. Ref. [20] summarized that cluster density is a composite phenotype formed by the coupling of multiple traits, such as pedicel length and branch length.

To address the lack of systematic integration of panicle economic traits, oil traits, and their association with quality in *I. polycarpa*, this study provides the first comprehensive analysis of materials from different regions by jointly evaluating panicle architecture, yield components, compactness phenotypes, and oil quality, focusing on panicle structure and branching traits, fruit set and single-fruit traits, as well as fatty-acid quality traits. Through trait correlation analysis, principal component analysis, and variation analysis, we identified key trait combinations affecting yield per panicle and panicle density, and clarified the yield-formation chain of “panicle architecture–branch differentiation–fruit number/fruit size.” We also analyzed oil content and differences among fatty-acid components to characterize the variation patterns of individual fatty-acid constituents [5,6,7]. These results provide a basis for establishing an evaluation system for panicle-related biological traits in *I. polycarpa,* support subsequent breeding studies, and offer phenotypic references for future QTL/GWAS mapping.

## 2. Results

### 2.1. Distribution Patterns of Panicle Structural Phenotypic Traits in I. polycarpa

**Figure 1:**
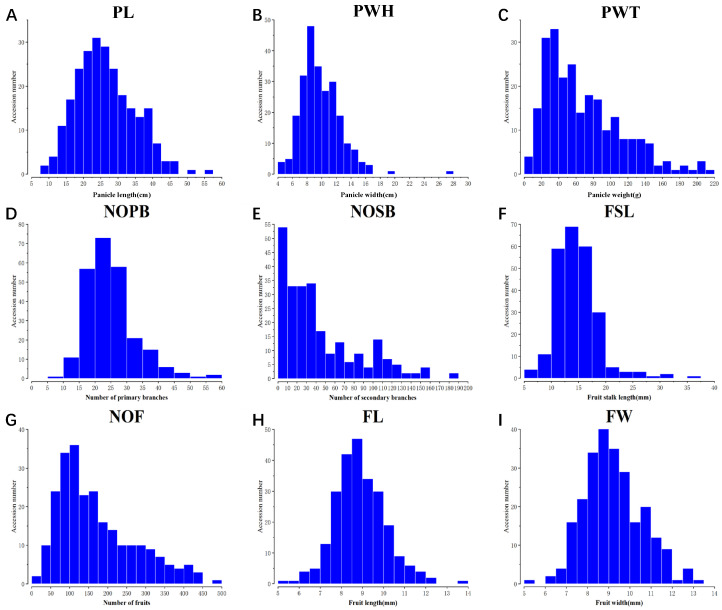
Frequency distributions of major panicle and fruit traits. (**A**–**I**) PL, panicle length; PWH, panicle width; PWT, panicle weight; NOPB, number of primary branches; NOSB, number of secondary branches; FSL, fruit stalk length; NOF, number of fruits; FL, fruit length; FW, fruit width.

Frequency distribution analyses were conducted for nine quantitative single traits in 250 *I. polycarpa* samples—PL, PWH, PWT, NOPB, NOSB, FSL, NOF, FL and FW (Figure 1). Overall, all traits showed continuous variation, consistent with the characteristics of typical quantitative traits. SL, NOPB, FSL, FL, and FW followed a normal or approximately normal distribution. In contrast, PWH, PWT, NOSB, and NOF exhibited log-normal/negative binomial distributions, indicating that the former group can be directly used for GWAS or QTL analyses, whereas the latter group shows pronounced right-skewness and should be log- or Box–Cox-transformed before GWAS or QTL mapping. Notably, NOSB and NOF displayed strongly right-skewed, long-tailed distributions in the population; NOSB also showed clear zero inflation and clustering at low values (NOSB = 0 accounted for 20%, and NOSB ≤ 10 accounted for approximately one-third), while extreme high values extended to around 200.

### 2.2. Correlations Among Major Panicle and Fruit Traits in I. polycarpa

Correlation analysis showed that the single-panicle yield indicator, PWT, was highly significantly and positively correlated with NOF and FWPHF, indicating that yield per panicle is mainly driven by NOF and FWT. NOF was positively correlated with the NOPB and NOSB, suggesting that fruit-bearing sites provided by secondary branches constitute an important structural basis determining fruit number. FWPHF was highly significantly and positively correlated with the fruit-size traits FL and FW. In contrast, FWPHF, FW, and FL showed weak correlations with NOF and with the numbers of primary and secondary branches; together, these three traits formed a relatively independent “fruit-size module” (Table 1). Overall, the results support decomposing yield-related traits into two separable phenotypic axes: “fruit-load output” (PWT/NOF/NOSB) and “fruit size” (FL/FW/FWPHF). Interestingly, FSL was associated with both modules.

### 2.3. Variation Analysis of Quantitative Traits in I. polycarpa

Variation analysis showed that the NOSB PWT and NOF exhibited high variation (CV = 60–95%), making them the most variable traits in the population. In particular, NOSB had the highest CV (93.95%). PL, NOPB, FWPHF, and FSL showed moderate variation (CV = 25–40%). In contrast, FL and FW displayed relatively low variation (CV < 15%), suggesting that fruit morphology is more stable within the population (Table 2). These results indicate that the genetic variation underlying single-panicle yield (PWT) is mainly associated with fruit-load traits such as branch number and NOF, especially secondary branching, followed by FWPHF, rather than fruit dimensions such as length and width.

### 2.4. Principal Component Analysis of Quantitative Traits in I. polycarpa

After standardizing the data for 10 panicle and fruit traits, principal component analysis (PCA) was performed. The results showed that the eigenvalues of the first five principal components were 3.87, 2.92, 0.91, 0.85 and 0.45, explaining 38.55%, 29.08%, 9.08%, 8.45% and 4.52% of the variance, with a cumulative explained variance of 89.69%; therefore, PC1–PC5 were retained for subsequent integrative analyses (Table 3). The loading matrix indicated that PC1 was mainly driven by PWT, NOF, PL, and PWH, reflecting integrated variation in overall panicle size and fruiting capacity. PC2 was mainly contributed by FL and FW and showed an opposing trend to branch number and NOF, representing a trade-off between fruit size and panicle architecture/fruit set. PC3 had the highest contribution from the NOPB, capturing differences in primary branching structure. PC4 was primarily influenced by FSL. PC5 was dominated by the NOSB and showed an opposite trend to PWH, suggesting a potential structural balance between secondary branching and lateral panicle expansion. The fact that FL/FW (fruit shape), NOPB, PL, and NOSB loaded primarily on PC2, PC3, PC4, and PC5, respectively, indicates that their genetic variation is largely independent, which is favorable for independent selection of breeding indices.

### 2.5. Correlation and Variation Analyses of Panicle Density Indices in I. polycarpa

**Figure 2:**
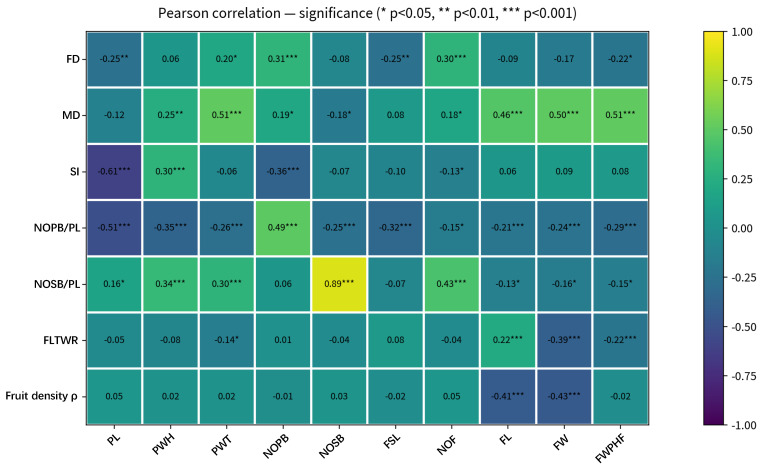
Significance analysis of correlations between indices and yield-related traits. *X*-axis: different traits; *Y*-axis: correlation coefficient.

Correlation analysis between the indices and yield-related traits (Figure 2) showed that fruit density per unit area was significantly negatively correlated with PL and FSL, whereas fruit mass per unit area (MD) was significantly positively correlated with fruit size and PWH. Notably, primary branch density was significantly negatively correlated with FSL and fruit size, while secondary branch density showed a significant positive correlation with NOF. Because fruit density and mass per unit area are key indicators for screening fruit rot rate, FSL, fruit size, and PWH are the main reference traits.

Variation analysis of the indices (Table 4) indicated that secondary branch density (NOSB/PL) had the highest coefficient of variation (CV, 87.77%), followed by mass density (CV = 46.67%) and fruit density (CV = 41.79%). This suggests that secondary branch density per unit length and load per unit length differ most markedly among accessions. Primary branch density (NOPB/PL) and the panicle shape index (SI) showed moderate variation (around 30%), reflecting certain differences in lateral panicle expansion and framework proportion. In contrast, the fruit shape index (FLTWR) exhibited the lowest variation (9.37%), indicating that fruit shape is relatively stable within the population. As a derived outcome based on the nine primary traits, the variation patterns of the density indices were generally consistent with those of the original nine traits.

### 2.6. Analysis of Fatty-Acid Component Traits in I. polycarpa

Analysis of fatty-acid component traits showed that IPFO is dominated by unsaturated fatty acids, with LA as the predominant component (54.86–71.99%, mean 64.00%), followed by PA (mean 20.43%) and POA (mean 7.50%). OA and ALA accounted for smaller proportions (means of 5.74% and 0.92%, respectively) (Table A1). Materials from different sampling sites differed markedly in fatty-acid relative proportions (%), absolute contents (mg/g), and oilPct (Table 5): oilPct ranged from 6.2% to 21% (mean 11.33%), and TotalFA ranged from 38.97 to 171.23 mg/g (mean ~82.42 mg/g).

**Figure 3:**
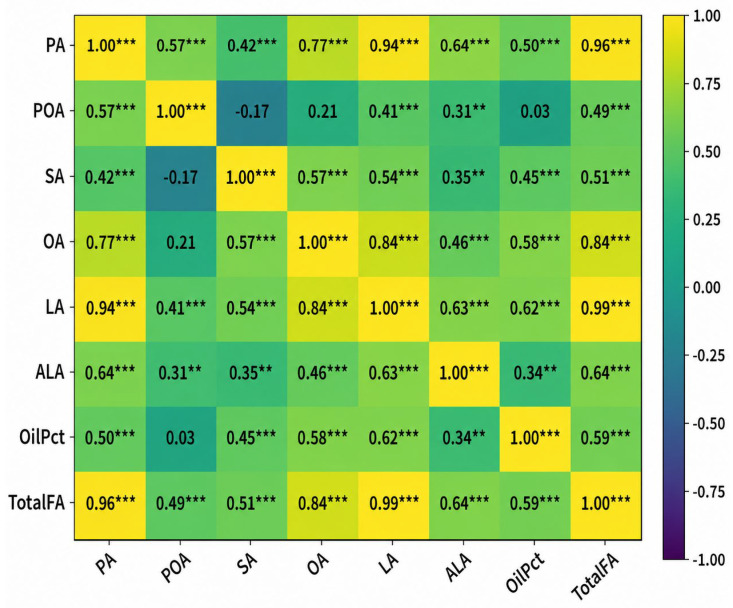
Correlation analysis among fatty-acid components significance. Significance: ** *p* < 0.01, *** *p* < 0.001.

**Figure A1:**
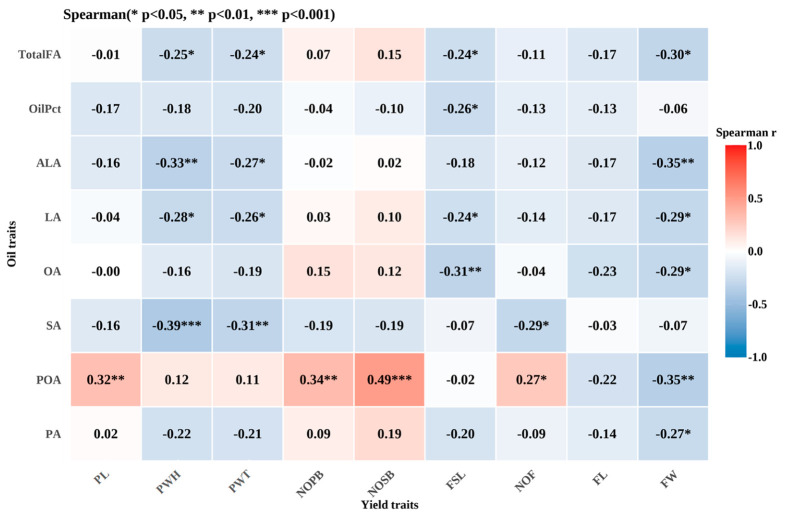
Correlation analysis between yield-related traits and absolute fatty-acid contents (mg/g), TotalFA, and oilPct.

Accession-level correlation analysis indicated that Pearson correlations among fatty-acid components were strong and significant, suggesting that the components vary synchronously and are mainly co-driven by the overall flux of total fatty-acid accumulation (Figure 3). In contrast, partial correlations among most fatty-acid components were weak (R^2^ < 0.05), implying that different components may be constrained by different rate-limiting enzymes and can therefore be selected relatively independently in breeding (Table A2). Further analysis showed no correlation with PWT (Figure A1), indicating that fatty-acid traits have independent breeding potential and can be jointly incorporated with yield/panicle-architecture traits into the phenotypic evaluation system to support the selection of high-yield, high-quality materials.

Variation analysis of absolute quantification for fatty-acid components showed that LA had the highest content (28.14–136.85 mg/g; mean 60.79 mg/g), followed by PA (4.31–22.84 mg/g; mean 11.02 mg/g), whereas SA and ALA were relatively low (means of 0.80 and 0.85 mg/g, respectively). In terms of coefficients of variation (CV), POA and OA exhibited the largest fluctuations (CV = 62.63% and 59.92%, respectively), followed by SA (50.82%). LA and PA showed moderate variation (44.92% and 42.82%), and the CV for TotalFA was 43.28%. By comparison, oilPct (6.20–21.00%; mean 11.33%) had a much lower CV of 26.69% (Table 6). These results indicate that overall fatty-acid content is relatively more stable, whereas the composition of fatty-acid components—especially minor constituents—varies more substantially, suggesting that fatty-acid component traits provide independent targets for improvement that can be selected in parallel with yield and panicle-architecture traits.

### 2.7. Correlation Analysis Between Fatty-Acid Traits and Yield-Related Traits in I. polycarpa

**Figure A2:**
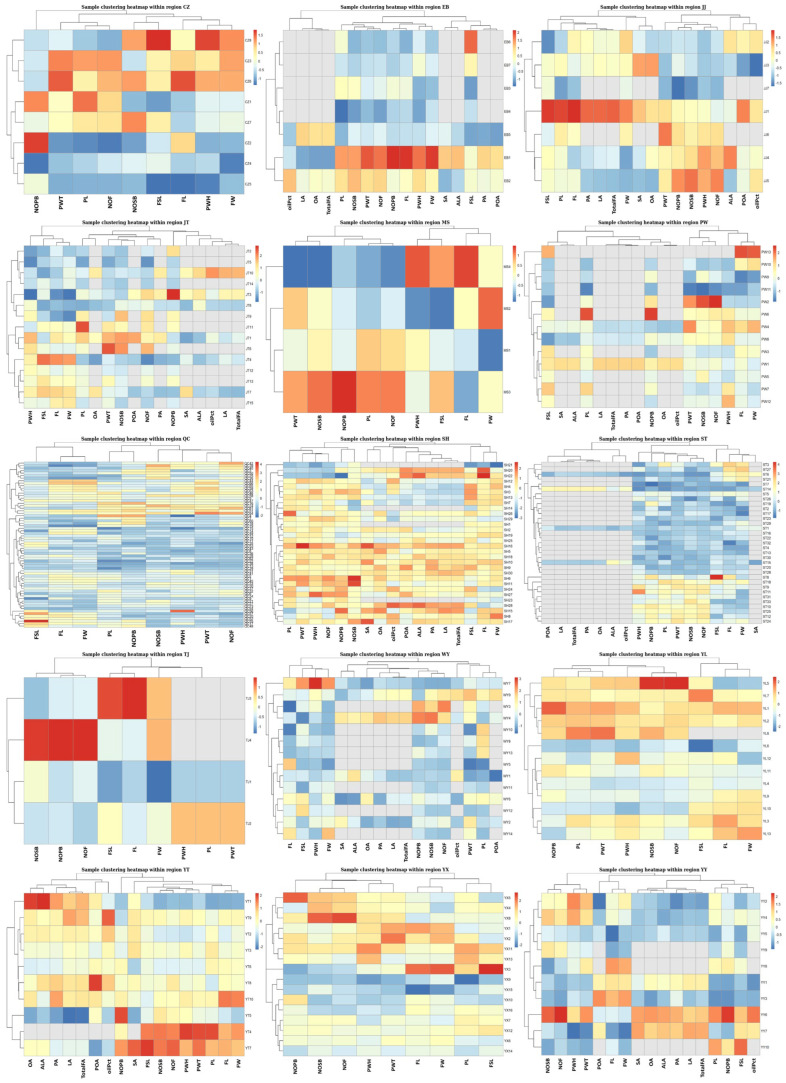
Clustered heatmap of sample traits within each population. *X*−axis: different traits; *Y*−axis: sampling points and their numbers.

After standardizing (Z-score) yield-component traits (NOPB, NOSB, NOF, and PWT/PL/PWH), fruit morphological traits (FSL, FL, and FW), and oil-quality traits available for some regions (oilPct, TotalFA, and major fatty-acid components), a clustered heatmap was generated for samples within each region (shown as a color heatmap, where red/orange indicates relatively higher trait values within the region, blue indicates relatively lower values, and gray denotes missing values) (Figure A2). The results showed that, in the EB region, samples represented by EB1 and EB2 exhibited a relatively balanced performance in both yield components and oil-related indices. In the JJ region, JJ1 and JJ4/JJ5 showed clear advantages in yield-component traits. In the YT region, YT7 (as well as YY6 from the Yanyuan, YY, region) displayed generally higher values across most traits, representing elite candidates with outstanding overall performance. YL1 and Jintang TJ4 also showed generally high performance in yield-related indicators. Notably, YY6 (YY) and JJ1 (JJ) performed better in oil-quality traits as well, representing comprehensive materials with both yield potential and quality advantages. These selections provide a set of candidate materials for subsequent utilization of elite germplasm and genetic mapping analyses.

**Figure 4:**
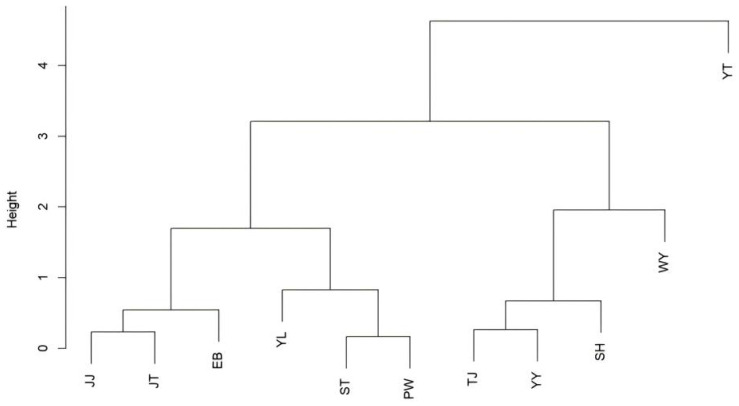
Population dendrogram based on the yield composite index and oilPct.

Clustering of the populations based on the “yield composite index (YieldIndex) + oilPct” could be summarized into three groups: a low-yield/low-oil type (JJ, JT, and EB clustered together), an intermediate type (YL clustered closely with ST, and PW), and a high-yield/high-oil type (TJ, YY, SH, and WY clustered together; among them, WY was slightly more distant from the other high-yield regions and showed a more pronounced yield advantage). In contrast, YT formed a separate branch with the greatest distance from the other regions, representing a typical high-oil but low-yield region (Figure 4).

**Figure A3:**
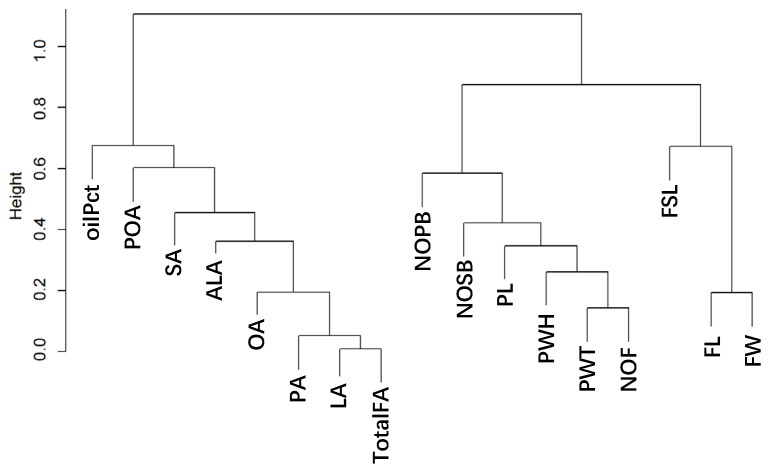
Trait dendrogram.

The traits could be grouped into three major modules: (i) oil/fatty-acid-related indices (oilPct, TotalFA, and individual fatty-acid components) clustered together, (ii) panicle yield-component traits (NOPB, NOSB, NOF, PL, PWH, and PWT) formed another cluster, and (iii) fruit morphological traits (FSL, FL, and FW) constituted a relatively independent cluster (Figure A3).

## 3. Discussion

### 3.1. Single-Panicle Yield in I. polycarpa Is Regulated by Hierarchical Primary/Secondary Branching Traits and Fruit-Weight Traits

Single-panicle yield in *I. polycarpa* is mainly determined by the product of NOF and single-fruit weight (primarily represented by FWPHF). Our results indicate that it is mainly driven by NOF, although FW should not be ignored. NOF is jointly determined by the NOPB, NOSB, and NOF directly borne on the main axis. We found that both NOF and NOSB exhibited significantly right-skewed, long-tailed distributions; the coefficient of variation (CV) further indicated that NOSB is a major source of variation in NOF. These results suggest that NOF is jointly constrained by primary and secondary branching architecture, and that the long tail and extreme high values of NOF are driven by an “amplification effect” of secondary branching. NOPB behaves as a relatively stable quantitative trait, providing the basic branching framework and an upper capacity limit, whereas NOSB further expands fruit-bearing sites on this basis. This implies that breeding for large-panicle phenotypes with high NOF should primarily target accessions with pronounced NOSB.

NOSB becomes a key trait because it simultaneously reflects two critical processes: whether secondary branches can initiate and, once initiated, how long they can continue to proliferate, thereby exerting an amplification effect on fruit number and single-panicle yield. Specifically, studies on *LAX1* indicate that secondary branching is initially constrained by the axillary or branch meristem initiation capacity; if this step is limited, the number of potential branch sites is significantly reduced [21]. On the other hand, studies on *TAW1* show that the number of branches also depends on the duration before meristems transition into spikelet meristems; when this phase transition is delayed, branch meristems can be maintained longer, producing more secondary branches [22]. Therefore, the pronounced variation in NOSB likely arises from the combined effects of these two developmental–genetic processes: meristem initiation capacity and meristem maintenance duration.

Research on rice panicle traits provides a valuable reference for *I. polycarpa*. In rice, panicle architecture/branching structure—the “branching system” formed by primary and secondary branches—makes a decisive contribution to grain number [23]. Primary and secondary branching may indeed be hierarchically regulated by different types of major-effect genes; therefore, using the numbers of primary branches, secondary branches, and their derived grain-number-related traits as targets for genetic dissection has a clear biological basis [23,24]. In *Arabidopsis*, shoot branching plays an important role in final yield formation, and *BRC1* has been identified as one of the key hubs in the branching regulatory network [25]. By decomposing NOF in *I. polycarpa* into primary and secondary branching components, our analyses indicate that fruit-bearing capacity in *I. polycarpa* is likewise subject to hierarchical regulation by primary and secondary branching.

NOSB exhibited a markedly right-skewed, long-tailed distribution, with clear zero inflation and clustering at low values. Subsampling analyses showed relatively small year-to-year variation in panicle traits, suggesting that NOSB may involve a “switch-like” genetic differentiation, in which some accessions are less likely to enter the developmental pathway of secondary-branch formation. This pattern is consistent with the discontinuous variation reported for branching-related traits in crops. For example, secondary branch number (SBN) shows discontinuous variation in some backcross populations and was inferred to be strongly controlled by a single recessive allele mapped to the *qSBN7* interval [26]. In our study, NOSB still displayed a continuous long tail beyond the low-value cluster, indicating that, once secondary branching is permitted, its extent is further regulated quantitatively. This pattern suggests that NOSB is jointly influenced by threshold-like control of branch initiation and by additive effects of multiple loci at the population level.

FWT was jointly determined by fruit volume and fruit density. Our results showed that FWT was closely associated with morphological traits such as FL and FW. FL and FW varied by 13–15%, whereas fruit volume varied by 32%; the estimated variation in fruit density was 18%, with an approximately normal distribution. In *I. polycarpa*, fruit density mainly reflects the volumetric proportion of pulp and seeds. Because the seeds contain bitter and other compounds and are removed during processing, fruit density may represent an important yield-related trait associated with seed-to-pulp proportion. The moderate variation in fruit volume also provides a basis for selecting large-fruited parents, while the weak association between fruit volume and density further supports the selection of large-fruit, small-seed (high pulp ratio) cultivars. Similar stability of size-related traits has also been reported in rice and wheat [27]. In addition, the “fruit-load output” module (PWT/NOF/NOSB) showed little correlation with the “fruit-size” module (FL/FW/FWPHF), indicating that these trait sets are relatively independent and may be improved simultaneously in breeding.

### 3.2. Panicle Density in I. polycarpa Is Jointly Regulated by Fruit Traits, Stalk Traits, Primary Branching, and Other Indices

In grapevine, the formation of the “loose–compact” cluster phenotype is determined by the overall berry volume (berry number × single-berry size/weight) and the rachis/branch framework, including the main axis, lateral axes/branches, and lengths and internode lengths of the cluster peduncle and pedicels [14]. We found that the key index for panicle density in *I. polycarpa*, MD, was closely associated with fruit-size traits such as FL and FW, whereas another major density index, FD, was associated with the NOPB and FSL. These results indicate that panicle density in *I. polycarpa* is shaped by factors similar to those governing grape cluster density.

However, in this study, the associations between these two density indices and other traits were generally weak, except for the strong relationship with fruit size; as a result, breeding high-panicle-weight *I. polycarpa* lines with moderate density still lacks clearly defined target traits. In practice, we identified heavy-panicle individuals with different “density” types within a set of half-sib families, indicating that high PWT and density are not inherently contradictory. Insights from studies on grape cluster density suggest that additional traits should be evaluated, such as the lengths of primary and secondary branchlets and the spacing between branchlets.

From a breeding perspective, these results suggest that NOSB may be prioritized as a structural target for selecting high-yield accessions, whereas MD can be used as a more comprehensive compactness-related indicator to balance fruit load and harvest performance. Therefore, breeding should prioritize accessions combining high NOSB and high PWT with moderate MD.

**Figure A4:**
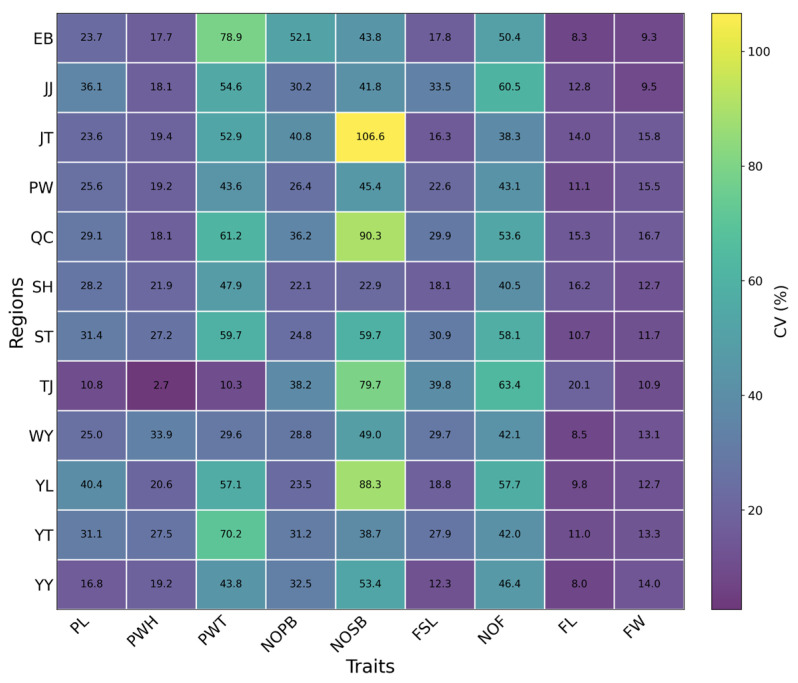
Heatmap of coefficients of variation (CVs) for major traits within each of the 12 regional populations.

Regional CV analysis further showed that substantial phenotypic variation was retained within each regional population (Figure A4). Branching- and fruit-load-related traits, especially NOSB, PWT, and NOF, generally exhibited higher within-region CV values than fruit-size traits such as FL and FW. This pattern indicates that phenotypic diversity in *I. polycarpa* is retained not only among regions but also within individual regional populations.

The substantial within-region variation observed for NOSB, PWT, and NOF suggests that each natural population still harbors exploitable variation for germplasm selection, and that elite accessions may be identified not only among regions but also within individual regions. By contrast, the relatively low within-region variation in FL and FW indicates that fruit-size traits are comparatively stable, which may facilitate their maintenance during selection. From a breeding perspective, selection for high-yield panicle architecture can therefore be conducted both across and within regions, especially by targeting highly variable traits such as NOSB and PWT.

### 3.3. The Relative Independence Between Fatty-Acid Traits and Yield-Related Traits Provides a Basis for Breeding Heavy-Panicle, High-IPFO

Fatty-acid traits and oilPct in *I. polycarpa* showed high levels of variation, with the ratios of the maximum to minimum values reaching 3.38–4.38, providing an important basis for selecting high-oil cultivars. In grape, seed oil content and composition vary greatly among cultivars, with oil content ranging from 4.03% to 18.01%, and it has been emphasized that oil traits are influenced by cultivar/accession as well as environmental management [28]. These findings indicate that, in perennial fruit trees, “oil traits” generally possess ample genetic variation for selection.

Fatty-acid levels and yield-related traits in *I. polycarpa* appeared to be relatively independent. Overall, oil-related traits showed weak correlations with yield-component indicators; for example, PWT and branching/fruiting traits (NOPB, NOSB, and NOF) had correlation coefficients of <0.3 with TotalFA and most major fatty-acid components. This suggests that lipid accumulation and yield formation do not vary along the same dominant axis. In rice, redirecting carbon flux from starch to lipids increased grain oil content to ~11.7%, in contrast to thousand-grain weight, seed-setting rate, and overall yield [29]. In *I. polycarpa*, however, such a trade-off was not observed as a systematic suppression of key panicle-yield traits. This further supports the view that oil level and yield components are not governed by the same major source of variation. Both individual-level analyses and population clustering identified accessions combining high yield (heavy panicles) with high oilPct (Figure 4), suggesting that *I. polycarpa* may possess genetic variation that partially decouples lipid accumulation from yield formation.

This relative independence is of direct relevance to breeding because it suggests that yield-related traits and oil-quality traits may be improved in parallel rather than through a strict trade-off. Moreover, recent work has shown that *I. polycarpa* oil by-products are rich in phospholipids enriched in polyunsaturated fatty acids, with potential applications in the food, pharmaceutical, and cosmetic industries [30], further highlighting the importance of improving both productivity and end-use quality simultaneously.

Some fatty-acid components showed weak negative correlations with panicle size and fruit size, indicating that breeding should avoid extreme single-trait selection that may inadvertently constrain size-related traits. Instead, multi-trait optimization based on “yield + oil” is recommended, with priority given to accessions that simultaneously exhibit high PWT and high oilPct.

## 4. Materials and Methods

### 4.1. Selection of Natural Populations of I. polycarpa and Distribution of Sampling Sites

**Figure A5:**
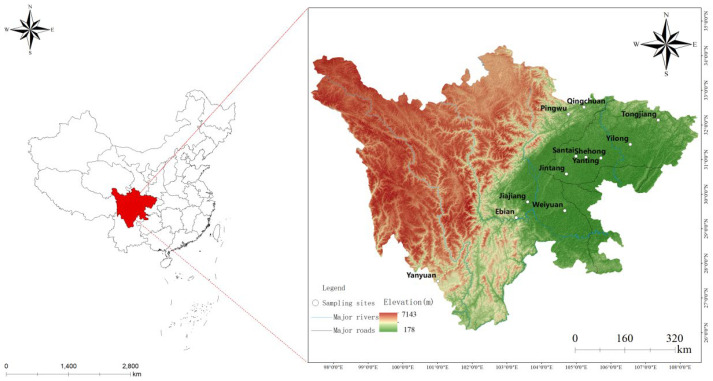
Schematic map of sampling site distribution.

The main natural distribution of *I. polycarpa* is in southwestern China, with a particular concentration in the Sichuan Basin and the surrounding mountainous areas. Therefore, sampling sites in this study were mainly located along the transitional ecological gradient of “basin–hills–mountains/plateau margin” in the Sichuan Basin and its peripheral highlands (Figure A5). In total, 12 sampling sites were included: Qingchuan (QC), Pingwu (PW), Tongjiang (TJ), Santai (ST), Yanting (YT), Shehong (SH), Jintang (JT), Yilong (YL), Weiyuan (WY), Jiajiang (JJ), Ebian (EB), and Yanyuan (YY). Sampled trees were required to exhibit vigorous natural growth, with no obvious pests or diseases, and to be at the young stage. The sampling sites spanned latitudes of 27.489–32.517° N, longitudes of 101.010–107.365° E, and elevations of 335–2705 m (Table A3), showing pronounced spatial and topographic heterogeneity and providing a diverse environmental background for analyzing variation in panicle and fruit traits of *I. polycarpa*.

### 4.2. Trait Measurement Methods

**Figure 5:**
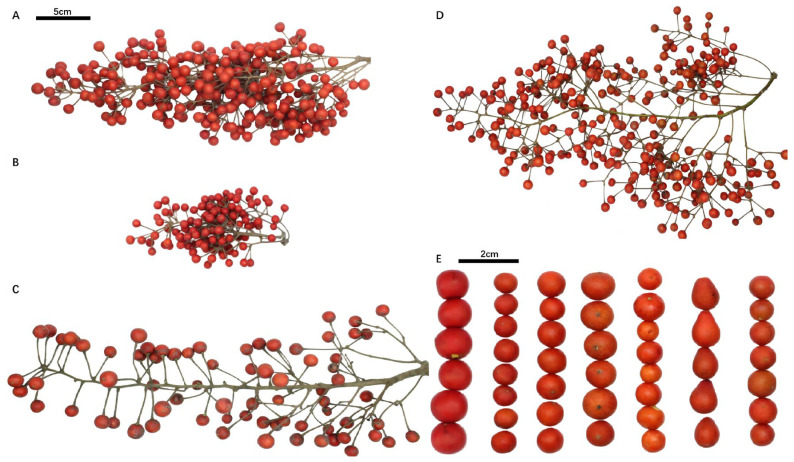
Phenotypes of *I. polycarpa.* (**A**): Long-panicle phenotype. (**B**): Short-panicle phenotype. (**C**): Phenotype with a high number of primary branches and long fruit stalk. (**D**): Phenotype with a high number of secondary branches and wide panicles. (**E**): Fruit phenotype.

Here, we investigated 250 individual accessions collected from natural populations across 12 regions, with a minimum distance of more than 20 m between sampled individuals within each region. For each accession, 3–6 panicles were collected. All traits were measured for each accession individually. Subsequent variation, correlation, and principal component analyses were performed using the combined accession-level dataset across all regions, rather than pooled regional means. Regional grouping was used only for population-level clustering and comparison analyses. In our preliminary investigations, substantial phenotypic variation was observed among different accessions in traits such as main panicle length, main panicle width, branch number, fruit stalk length, fruit number, and panicle weight (Figure 5). Therefore, nine major panicle-related quantitative traits were measured: panicle length (PL), panicle width (PWH), panicle weight (PWT), number of primary branches (NOPB), number of secondary branches (NOSB), fruit stalk length (FSL), number of fruits (NOF), fruit length (FL), and fruit width (FW).

PL and PWH were measured using a ruler (to 0.1 cm). PWT was measured with an electronic balance (to 0.01 g). FSL, FL, and FW were measured with a digital vernier caliper (15 replicates per panicle), and calculated fruit weight per hundred fruits (FWPHF = PWT/NOF) as a composite trait. The remaining traits were recorded by direct counting. To characterize panicle density, indices were constructed based on the raw traits by following the quantitative approach used for grape cluster density: (1) fruit density, FD = NOF/SA (panicle area) (fruits/cm^2^); (2) mass density, MD = PWT/SA (g/cm^2^); (3) shape index, SI = PWH/PL; (4) primary and secondary branch densities, NOPB/PL and NOSB/PL, respectively; and (5) fruit shape index (FLTWR = FL/FW) and fruit density, ρ = single-fruit weight/volume (g/cm^3^, volume v = π × FL × FW^2^/6). These derived indices were used to describe fruit crowding, mass load, and branching configuration per unit projected area or per unit axis length of the panicle.

**Figure 6:**
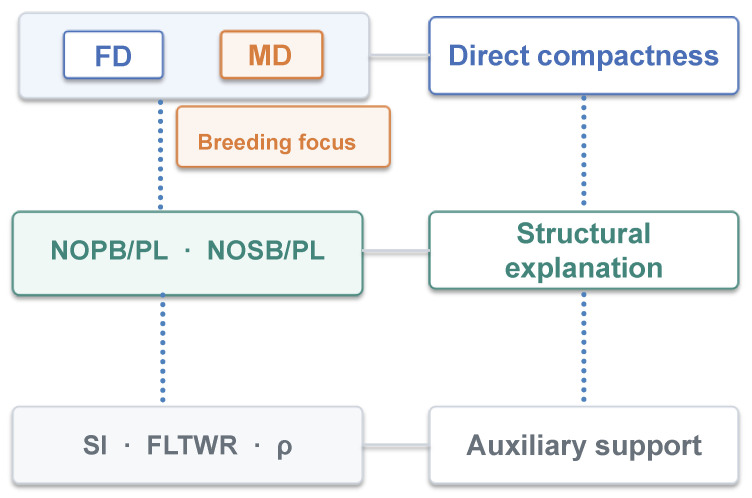
Schematic diagram of relationships among density indices.

Here, the derived indices were classified into three categories. FD and MD are direct compactness indices, where FD represents the degree of fruit crowding per unit projected area, and MD represents the fresh weight load per unit projected area. Compared with FD, MD integrates both fruit number and fruit weight, serving as a comprehensive indicator of panicle compactness and its potential harvest risk. NOPB/PL and NOSB/PL are structural explanatory indices, describing the intensity of branching configuration per unit panicle length. SI, FLTWR, and ρ are defined as auxiliary morphological indices, providing supplementary descriptions of compactness phenotypic variation (Figure 6).

### 4.3. Determination of Fatty-Acid Components in I. polycarpa

**Figure A6:**
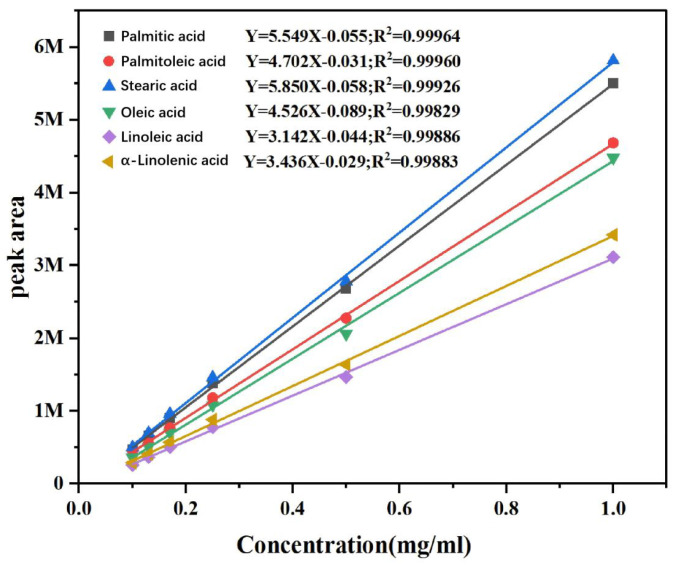
External standard calibration curves for fatty acids. *X*-axis: sample concentration; *Y*-axis: peak area. The regression equation and R2 are labeled on the plot.

After comprehensively considering factors such as sample maturity, post-harvest handling, and storage, a total of 71 high-yielding accessions from different regional populations were selected for fatty-acid analysis to examine the relationship between fatty-acid content and yield-related traits. Fatty-acid determination followed GB 5009.168—2016 [31]. Fatty-acid methyl esters (FAMEs) were separated and quantified by gas chromatography. Reagents including 8.3 mol/L HCl, diethyl ether–petroleum ether (1:1, *v*/*v*), 2% NaOH–methanol, and saturated NaCl solution were prepared. Fruit pulp powder of *I. polycarpa* (0.5 g), ground in liquid nitrogen and stored at −80 °C, was used as the sample. After acid hydrolysis (70–80 °C water bath for 40 min), lipids were extracted with diethyl ether/petroleum ether, and the extract was rotary-evaporated and weighed. The oil was then subjected to saponification and BF_3_–methanol methylation. Following extraction with n-heptane, drying over anhydrous sodium sulfate, and filtration through a 0.22 μm membrane, FAMEs were analyzed using a Varian 450-GC equipped with a 60 m × 0.25 mm × 0.5 μm capillary column under the following conditions: injector temperature, 270 °C; detector temperature, 280 °C; oven temperature program, 50 °C to 230 °C at 20 °C/min with a final hold of 22 min; nitrogen as the carrier gas; split ratio, 20:1; injection volume, 1.0 μL. External standard calibration curves were established using standards of palmitic acid (PA), palmitoleic acid (POA), stearic acid (SA), oleic acid (OA), linoleic acid (LA), and α-linolenic acid (ALA) (Figure A6). The coefficients of determination (R^2^) for all curves were above 0.998, meeting the requirements for quantitative analysis. The sum of these six major fatty acids was calculated as total fatty-acid content (TotalFA), and oil content (oilPct) was calculated as the mass of extracted oil divided by the sample mass (0.5 g).

### 4.4. Data Analysis Methods

Sampling-site maps were generated using ArcMap 10.8. Microsoft Excel was used to calculate fruit density (NOF/panicle area), mass density (PWT/panicle area), panicle shape index (PWH/PL), primary branch density (NOPB/PL), secondary branch density (NOSB/PL), and fruit shape index (FL/FW). Phenotypic data were analyzed in SPSS 23 and visualized in Origin 2025. Descriptive statistics (mean, standard deviation, range, and coefficient of variation, CV) were computed for each trait to evaluate population-level variation. One-way analysis of variance (ANOVA) was used to compare trait differences among sampling sites; Pearson correlation analysis was applied to examine trait associations; and principal component analysis (PCA) was conducted to extract integrated trait dimensions and quantify trait contributions.

## 5. Conclusions

This study established a hierarchical regulatory framework for economically important panicle traits in *I. polycarpa*, clarifying that yield per panicle is jointly determined by branching architecture, fruit-number formation, and FWT. Analyses of trait distributions, correlations, and variation patterns indicated that NOF is constrained by both primary and secondary branching. Primary branches mainly provide the structural framework of the panicle, whereas secondary branches play dual roles in “switch-like differentiation” and “quantitative amplification,” making them a key structural trait driving extreme high values and expanded variation in NOF. Therefore, secondary branching can be prioritized as a selection target for breeding heavy-panicle, high-yield ideotypes. Differences in FWT are jointly regulated by fruit volume and fruit density, providing a basis for selecting processing-oriented traits such as “large fruit with small seeds (high pulp ratio).”

Panicle compactness, an important trait affecting the proportion of marketable fruits at harvest, was mainly influenced by fruit size, FSL, and primary branching architecture. Further evaluation should incorporate additional traits such as branchlet length and branchlet spacing to improve assessment accuracy. oilPct and fatty-acid traits showed overall weak correlations with yield-component traits, indicating that high-yield and high-oil traits have relatively independent potential for improvement, thereby providing a basis for joint breeding of “heavy-panicle, high-oil” ideotypes. Overall, this study provides a phenotypic foundation for integrated selection of panicle yield, density, and oil-quality traits in *I. polycarpa*, and also offers key traits and modeling directions for subsequent GWAS and molecular breeding research.

## Figures and Tables

**Table 1:** Correlations among phenotypic traits in *I. polycarpa*.

Trait	PWH	PWT	NOPB	NOSB	FSL	NOF	FL	FW	FWPHF
PL	0.58 ***	0.68 ***	0.52 ***	0.48 ***	0.47 **	0.70 ***	0.09	0.11	0.14 *
PWH		0.76 ***	0.27 ^+^	0.61 ***	0.40 *	0.72 ***	0.18	0.21	0.24 ^+^
PWT			0.45 ***	0.57 ***	0.29 ^+^	0.86 ***	0.33 ***	0.36 **	0.43 **
NOPB				0.23 ^+^	0.05	0.60 ***	−0.10	−0.14	−0.14
NOSB					0.13	0.65 ***	−0.09	−0.05	−0.05
FSL						0.16	0.35 *	0.31 *	0.35 *
NOF							−0.09	−0.09	−0.06
FL								0.81 ***	0.81 ***
FW									0.87 ***

FWPHF is a derived index of PWT and NOF; significance: ^+^ *p* < 0.1, * *p* < 0.05, ** *p* < 0.01, *** *p* < 0.001.

**Table 2:** Variation analysis of phenotypic traits in *I. polycarpa*.

Trait	PL	PWH	PWT	NOPB	NOSB	FSL	NOF	FL	FW	FWPHF
Min.value	9.45	4.65	5.78	8.5	0	6.27	14.67	5.21	5.22	15.25
Max.value	55.61	27.00	211.84	57.67	186	36.86	592	13.75	13.39	100.99
Mean value	26.57	9.78	69.02	24.58	43.37	14.80	173.08	8.91	9.19	41.07
SD	8.53	2.73	44.82	7.93	40.75	4.00	104.65	1.24	1.38	15.22
CV/%	32.1	27.98	64.93	32.28	93.95	27.03	60.46	13.92	14.97	37.06

**Table 3:** Principal component analysis of phenotypic traits in *I. polycarpa*.

Trait		PL	PWH	PWT	NOPB	NOSB	FSL	NOF	FL	FW	FWPHF	Eigen Value	Contribution Rate/%	Cumulative Contribution Rate/%
Principal component	PC1	0.39	0.41	0.46	0.19	0.30	0.24	0.38	0.19	0.21	0.23	3.87	38.55	38.55
	PC2	−0.16	−0.05	−0.03	−0.24	−0.28	0.20	−0.32	0.48	0.48	0.48	2.92	29.08	67.64
	PC3	0.25	−0.27	−0.12	0.75	−0.40	0.33	−0.08	0.07	−0.01	−0.06	0.91	9.08	76.7
	PC4	0.18	0.20	−0.28	−0.36	0.08	0.77	−0.16	−0.11	−0.24	−0.15	0.85	8.45	85.17
	PC5	−0.35	0.51	0.21	−0.02	−0.68	0.06	0.22	−0.18	−0.12	−0.02	0.45	4.52	89.69

**Table 4:** Variation analysis of each index.

Indices	FD	MD	SI	NOPB/PL	NOSB/PL	FLTWR	ρ
Min.value	0.27	0.08	0.12	0.40	0.00	0.80	0.55
Max.value	2.38	1.10	0.88	2.43	6.82	1.41	1.89
Mean value	0.99	0.39	0.39	0.98	1.56	0.97	1.03
SD	0.41	0.18	0.11	0.33	1.37	0.09	0.19
CV/%	41.79%	46.67%	28.15%	34.02%	87.77%	9.37%	18.74%

**Table 5:** Ranges of absolute fatty-acid contents (mg/g) and TotalFA across different sampling sites.

Code	Location	Fatty-Acid Content (mg/g)						TotalFA (mg/g)
		PA	POA	SA	OA	LA	ALA	
JT	Jintang, Chengdu	8.33–11.72	1.77–6.44	0.47–0.98	1.41–3.63	38.26–76.91	0.66–1.26	53.91–96.83
ST	Santai, Mianyang	4.31–10.42	0.39–1.71	0.99–2.02	1.69–5.46	29.00–73.10	0.61–0.88	38.97–92.76
TJ	Tongjiang, Bazhong	7.03	3.06	0.45	1.92	35.23	0.38	48.07
EB	Ebian, Leshan	6.56–6.75	3.51–5.67	0.36–0.38	2.06–2.95	31.09–36.70	0.48–0.72	46.66–50.22
WY	Weiyuan, Neijiang	5.86–9.07	0.65–3.43	0.43–0.80	1.47–2.92	28.14–57.12	0.54–1.11	39.43–73.17
YY	Yanyuan, Liangshan	4.57–13.67	1.44–7.32	0.35–1.73	2.31–9.90	30.14–87.74	0.40–1.15	40.59–117.50
PW	Pingwu, Mianyang	6.28–14.26	1.84–9.27	0.36–0.78	2.10–6.49	32.41–63.44	0.53–0.93	43.88–95.17
YL	Yilong, Nanchong	5.52	2.70	0.56	1.84	32.65	0.74	44.00
JJ	Jiajiang, Leshan	6.40–15.02	1.16–5.40	0.36–1.03	1.74–4.32	29.84–69.36	0.39–1.75	41.60–95.25
YT	Yanting, Mianyang	9.75–22.84	1.44–6.55	0.63–1.98	3.19–14.45	43.29–136.85	0.75–1.60	61.33–171.23
SH	Shehong, Suining	5.69–18.41	2.22–12.29	0.40–1.13	2.05–6.50	31.49–99.18	0.43–1.46	46.27–132.51
total		4.31–22.84	0.39–12.29	0.35–2.02	1.41–14.45	28.14–136.85	0.38–1.75	38.97–171.23
average		11.02	4.74	0.80	4.23	60.79	0.85	82.42

**Table 6:** Variation analysis of absolute fatty-acid contents (mg/g), TotalFA, and oilPct.

Trait	PA	POA	SA	OA	LA	ALA	TotalFA	oilPct (%)
Min.value	4.31	0.39	0.35	1.41	28.14	0.38	38.97	6.2
Max.value	22.84	12.29	2.02	14.45	136.85	1.75	171.23	21
Mean value	11.02	4.74	0.8	4.23	60.79	0.85	82.42	11.33
SD	4.72	2.97	0.41	2.54	27.31	0.3	35.67	3.02
CV/%	42.82	62.63	50.82	59.92	44.92	35.52	43.28	26.69

## Data Availability

The data presented in this study are available on request from the corresponding author. The data are not publicly available due to privacy and ethical restrictions.

## Appendix A

**Table A1:** Ranges of fatty-acid composition (%) and oilPct across different sampling sites.

Code	Location	Fatty-Acid Composition (%)						oilPct (%)
		PA	POA	SA	OA	LA	ALA	
JT	Jintang, Chengdu	18.92–25.17	3.49–11.74	0.76–2.50	2.19–7.63	56.75–71.31	0.73–1.43	6.2–17
ST	Santai, Mianyang	15.37–18.53	0.90–3.16	2.09–6.83	3.81–7.12	65.39–71.99	0.68–1.81	10.8–12
TJ	Tongjiang, Bazhong	22.2	8.15	1.05	4.19	63.89	0.52	10.6
EB	Ebian, Leshan	19.89–21.41	8.05–15.36	0.72–0.81	4.57–6.62	56.64–63.99	0.71–1.20	9.4–10.6
WY	Weiyuan, Neijiang	17.55–24.08	1.75–7.19	0.93–2.53	3.22–6.09	61.86–69.52	0.80–2.23	7–13.4
YY	Yanyuan, Liangshan	16.82–22.98	4.19–16.51	0.69–2.30	4.35–10.84	54.86–65.98	0.55–0.89	10.2–12.6
PW	Pingwu, Mianyang	19.90–22.09	5.32–12.19	0.72–1.45	5.15–9.30	56.02–64.90	0.79–1.32	10.8–11.8
YL	Yilong, Nanchong	19.18	7.93	1.57	4.39	65.54	1.38	11
JJ	Jiajiang, Leshan	21.01–24.49	2.21–8.77	0.86–2.28	3.63–7.86	61.24–64.39	0.63–2.95	8.2–9
YT	Yanting, Mianyang	16.81–23.82	1.55–7.64	0.89–2.61	5.79–10.33	60.48–71.79	0.51–0.98	14.2–21
SH	Shehong, Suining	18.40–24.18	5.83–16.88	0.53–1.89	2.85–7.36	54.96–68.12	0.51–1.20	6.6–15.2
total		15.37–25.17	0.90–16.88	0.53–6.83	2.19–10.84	54.86–71.99	0.51–2.95	6.2~21
average		20.43	7.5	1.41	5.74	64	0.92	11.33

**Table A2:** Partial correlation analysis among fatty-acid components.

Trait	PA	POA	SA	OA	LA	ALA
PA	1.00	0.31	0	0.01	0.56	0.04
POA	0.31	1.00	0.15	0.04	0.03	0
SA	0	0.15	1.00	0.02	0.04	0.01
OA	0.01	0.04	0.02	1.00	0.16	0.03
LA	0.56	0.03	0.04	0.16	1.00	0.01
ALA	0.04	0	0	0.03	0.01	1.00

**Table A3:** Summary of sampling-site latitude/longitude coordinates and elevations.

Location	Latitude (° N)	Longitude (° E)	Elevation (m)
Jiajiang, Leshan	29.777197	103.595184	421
Ebian, Leshan	29.327914	103.264443	958
Santai, Mianyang	31.100267	104.992759	417
Qingchuan, Guangyuan	32.516917	105.21916	816
Pingwu, Mianyang	32.308568	104.77602	831
Jintang, Chengdu	30.585213	104.717974	460
Yilong, Nanchong	31.438921	106.559006	421
Yanyuan, Liangshan	27.48916	101.010188	2705
Yanting, Mianyang	31.044268	105.708989	438
Shehong, Suining	31.067489	105.277749	391
Weiyuan, Neijiang	29.527448	104.669113	335
Tongjiang, Bazhong	32.142595	107.365351	559
